# Effect of cardiac rehabilitation on cognitive function in elderly patients with cardiovascular diseases

**DOI:** 10.1371/journal.pone.0233688

**Published:** 2020-05-29

**Authors:** Kazuhiro Fujiyoshi, Yoshiyasu Minami, Minako Yamaoka-Tojo, Toshiki Kutsuna, Shinichi Obara, Akihiro Aoyama, Junya Ako

**Affiliations:** 1 Department of Cardiovascular Medicine, Kitasato University School of Medicine, Sagamihara, Japan; 2 Department of Rehabilitation, Kitasato University School of Allied Health Sciences, Sagamihara, Japan; 3 Department of Cardiac Rehabilitation, Kitasato University East Hospital, Sagamihara, Japan; Nathan S Kline Institute, UNITED STATES

## Abstract

**Background:**

Cognitive function is an important factor for secondary prevention in elderly patients with cardiovascular diseases. The objective of this study was to evaluate the impact of cardiac rehabilitation (CR) on the improvement of cognitive function.

**Methods:**

A total of 66 consecutive elderly patients (≥70 years old) with cardiovascular diseases were prospectively enrolled. The change in cognitive function during 6 months was compared between the patients with monthly CR (at least once per month; n = 27) and those without monthly CR (n = 39). Cognitive function was evaluated using the Mini-mental State Examination (MMSE) and Frontal Assessment Battery (FAB).

**Results:**

There was no significant difference in baseline characteristics between the 2 groups. The change in the MMSE score was significantly greater in patients with monthly CR than in those without monthly CR (2.3 ± 0.4 vs. −0.1 ± 0.3 points; *p* <0.001). Among the MMSE items, the change in temporal orientation and attention and calculation was significantly greater in the monthly CR group than in the non-monthly CR group (0.8 ± 0.7 vs. −0.1 ± 0.8 points [*p* <0.001] and 1.0 ± 1.5 vs. −0.1 ± 0.1 points [*p* <0.001], respectively). The general linear model revealed that monthly CR (effect estimate, 1.455; 95% confidence interval, 0.747–2.163; *p* <0.001) was independently associated with the change in the MMSE score.

**Conclusions:**

Cognitive function may improve with regular CR. These results might partly explain the efficacy of CR for secondary prevention.

## Introduction

An association between the onset of cardiovascular disease (CVD) and cognitive impairment in elderly patients through the so-called heart–brain continuum hypothesis has been proposed. [1] The presence or onset of CVD impairs cognitive function through the activation of a thrombotic state, cardiac dysfunction, and vascular endothelial dysfunction. [2] Conversely, the presence of cognitive impairment may worsen the status of CVD through insufficient medicine compliance, poor nutritional status, and limited physical activities. [3] Thus, the maintenance of cognitive function is an important factor for secondary prevention, and can be a therapeutic and interventional target in elderly patients with CVD. Previous studies have reported the efficacy of comprehensive rehabilitation including dietary therapy, exercise, and cognitive training on the improvement of cognitive function in patients with cognitive impairment. [4, 5] However, the efficacy of cardiac rehabilitation (CR) in improving cognitive function remains unclear. Therefore, in the present study, we investigated the change in cognitive function during the CR period and evaluated the efficacy according to the frequency of CR in elderly patients with CVD.

## Materials and methods

### Study population

This study was a prospective observational study conducted from October 2015 to June 2016. A total of 84 patients with CVD, who were enrolled in the CR program of Kitasato University East Hospital, were identified. CVDs included ischemic heart disease, atrial fibrillation, and chronic heart failure. A total of 18 patients were excluded because of age (<70 years), use of medications for dementia, refusal to participate in the study, or loss to follow-up. Finally, we included 66 patients in the analysis (S1 Fig). The study protocol was approved by the Kitasato University Medical Ethics Organization (KMEO B15-68) and conformed to the ethical guidelines of the Declaration of Helsinki. All patients signed the written informed consent form before participation.

### Assessment of cognitive function

Cognitive function was assessed using the Mini-mental State Examination (MMSE) and Frontal Assessment Battery (FAB) at baseline and at 6 months follow-up. The MMSE is a test evaluating orientation, registration, recording, writing, visual construction, and other items related to cognitive function, based on a 30-point scale. [6] The cognitive status were evaluated validated criteria. [7, 8] Cognitive improvement was defined as ΔMMSE > 0 point. [9] The FAB is a cognitive function test designed to evaluate the function of the prefrontal cortex based on an 18-point scale. [10] The investigators for MMSE and FAB measurements were blinded to the patients’ baseline clinical characteristics.

### Comprehensive CR program

The comprehensive CR program at the Center for Cardiovascular Disease Prevention, Kitasato University East Hospital included exercise and education, as follows: combined exercise with stretching, resistance, and aerobic training; education to increase physical activities; dietary therapy to improve coronary risk factors; and disease management to prevent atherosclerosis progression and cardiac events. [11, 12] The patients were encouraged to join an on-site supervised aerobic exercise session for 30 min at least once per month according to international guidelines. [13]

### Measurement of endothelial function

The reactive hyperemia peripheral arterial tonometry (RH-PAT) index was measured as a surrogate for endothelial function, by using a finger plethysmograph (EndoPAT2000; Itamar Medical, Caesarea, Israel). We measured the digital pulse amplitudes in patients while they were in the supine position for 5 min at baseline and after the induction of reactive hyperemia by means of forearm cuff occlusion for 5 min. [2] Data were digitized and computed automatically using the EndoPAT2000 software. The RH-PAT index was defined as the ratio of the mean post-deflation signal (in the 90–120 s post-deflation interval) to the baseline signal in the hyperemic finger, and was normalized using the same ratio in the contralateral finger. This value was multiplied by a baseline correction factor calculated using the EndoPAT2000 software. The detailed methods and definitions of the present study are described in Supplementary methods (S1 File).

### Measurement of physical function

Physical functions were measured at baseline and at 6 months follow-up. Quadriceps isometric strength was measured with a hand-held dynamometer (μ-Tas; ANIMA, Tokyo, Japan). With the patient in sitting position, 5-s maximal isometric voluntary contractions of the quadriceps were collected for both legs. The strength values on the both sides were averaged and expressed as absolute value (kg). [14] The 6-min walk distance was measured according to standard guidelines established by the American Thoracic Society. [15] Further details are described in Supplementary methods (S1 File).

### Definition

Ischemic heart disease included myocardial infarction, angina pectoris and vasospastic angina. Atrial fibrillation included both paroxysmal and chronic. Heart failure included both systolic dysfunction and diastolic dysfunction. Hypertension was defined as arterial blood pressure > 140/90 mmHg or taking antihypertensive medication. Other definitions are described in Supplementary methods.

### Statistical analysis

Normally distributed continuous variables were presented as means ± standard deviations, and continuous variables deviating from the normal distribution were presented as means and interquartile ranges. Comparison of variables between baseline and 6 months after treatment was done using the paired *t*-test or Wilcoxon signed-rank test. Student’s *t*-tests or Mann–Whitney *U*-tests were used to examine variables between the monthly CR group and the non-monthly CR group. Binary variables were presented as percentages and examined using chi-square tests. A general linear model with multiple predictor variables was used to determine independent clinical predictors of absolute changes in the MMSE and FAB scores. The receiver-operating characteristic (ROC) curve was constructed to assess the number of CR session needed to detect cognitive improvement evaluated using MMSE and FAB. A statistically significant difference was considered when the *p* values were ≤0.05. Statistical analyses were performed using JMP 9.0 version (SAS Institute, Cary, NC, USA).

## Results

### Comparison of baseline characteristics between monthly CR and non-monthly CR

Of 66 patients, 27 (41%) were allocated to the monthly CR group and 39 (59%) were allocated to the non-monthly CR group. There was no significant difference in the MMSE and FAB scores at baseline between the 2 groups. There was also no significant difference in other baseline clinical characteristics and physical function parameters between the 2 groups (Table 1).

**Table 1:** Baseline patient characteristics.

Variables	All patients n = 66	Monthly CR n = 27	Non-monthly CR n = 39	*p* value
***Clinical characteristics***				
Age, years	77.1 ± 4.7	78.2 ± 4.5	76.3 ± 4.8	0.105
Male, n (%)	37 (56)	17 (62)	20 (51)	0.345
Body mass index, kg/m^2^	23.6 ± 3.4	22.9 ± 3.7	24.0 ± 3.2	0.229
Education, years	12.5 ± 1.6	12.5 ± 1.8	12.5 ± 1.5	0.962
Mean Blood pressure, mmHg	89.3 ± 11.0	91.4 ± 13.8	87.8 ± 8.7	0.196
Myocardial infarction, n (%)	19 (29)	8 (29)	11 (28)	0.900
Atrial fibrillation, n (%)	22 (33)	6 (22)	16 (41)	0.106
Paroxysmal, n (%)	2 (3)	0 (0)	2 (5)	0.232
Chronic, n (%)	20 (30)	6 (22)	14 (35)	0.234
Heart failure, n (%)	22 (33)	9 (33)	13 (33)	1.000
Systolic, n (%)	8 (12)	2 (7)	6 (15)	0.328
Diastolic, n (%)	14 (21)	7 (25)	7 (25)	0.435
Hypertension, n (%)	59 (89)	25 (92)	34 (87)	0.474
Hyperlipidemia, n (%)	55 (83)	20 (74)	35 (89)	0.095
Diabetes mellitus, n (%)	23 (34)	11 (40)	12 (30)	0.404
Blood glucose, mg/dL	115 ± 18.4	111 ± 12.6	118 ± 21.6	0.156
Hemoglobin A1c, %	6.39 ± 1.00	6.64 ± 1.25	6.22 ± 0.77	0.093
LDL-cholesterol, mg/dL	97.5 ± 23.3	97.8 ± 21.9	97.3 ± 24.6	0.935
HDL-cholesterol, mg/dL	59.7 ± 14.1	59.5 ± 16.5	59.8 ± 12.5	0.932
Triglyceride, mg/dL	106.5 [76.5–140.0]	119.0 [77.0–199.0]	100.0 [75.0–134.0]	0.113
Brain natriuretic peptide, pg/mL	73.5 [39.1–123.7]	99.1 [46.1–140.0]	58.5 [35.5–108.7]	0.116
Left ventricular ejection fraction, %	61.0 ± 8.5	61.2 ± 8.9	60.8 ± 8.3	0.880
E/e′	13.1 ± 4.0	13.6 ± 4.5	12.8 ± 3.4	0.470
Intima media thickness, mm	2.3 ± 0.8	2.3 ± 0.7	2.2 ± 0.7	0.628
Pulse wave velocity, cm/s	1750 ± 319	1818 ± 301	1703 ± 326	0.152
RH-PAT index	1.74 ± 0.52	1.85 ± 0.65	1.67 ±0.41	0.160
MMSE, points	25.3 ± 2.8	25.2 ± 2.9	25.3 ± 2.7	0.929
Normal (MMSE >27), n (%)	15 (22)	7 (25)	8 (20)	0.792
Cognitive impairment without dementia (23< MMSE ≤27), n (%)	35 (53)	13 (48)	22 (56)	
Mild degree dementia (MMSE ≤23), n (%)	16 (24)	7 (25)	9 (23)	
FAB, points	13.5 ± 2.2	13.9 ± 1.9	13.2 ± 2.3	0.223
***Physical functions***				
Grip, kg	24.1 ± 9.9	24.1 ± 9.9	25.1 ± 9.3	0.695
Quadriceps isometric strength, kg	30.3 ± 9.9	30.3 ± 9.9	30.8 ± 9.4	0.849
Walking speed, m/s	1.60 ± 0.34	1.60 ± 0.34	1.69 ± 0.37	0.353
One-leg standing, s	38.2 ± 23.5	38.2 ± 23.5	32.2 ± 23.5	0.286
Functional reach, cm	34.6 ± 5.8	34.6 ± 5.8	37.0 ± 4.6	0.062
Six-minute walking distance, m	465 ± 97.5	465 ± 97.5	456 ± 85.1	0.679

Values are presented as mean ± standard deviation, or n (%). CR, cardiac rehabilitation; LDL, low-density lipoprotein; HDL, high-density lipoprotein; E/e′, peak early diastolic velocity/basal septal diastolic velocity ratio; RH-PAT, reactive hyperemia peripheral arterial tonometry; MMSE, Mini-mental State Examination; FAB, Frontal Assessment Battery.

### Comparison of the changes in laboratory findings and physical function between the monthly CR and non-monthly CR groups

The changes in laboratory findings and physical function are shown in Table 2. There was no cardiovascular re-hospitalizations during the study period in both groups. The decrease in hemoglobin A1c, triglyceride, and brain natriuretic peptide level was greater in the monthly CR group than in the non-monthly CR group. The change in physical function parameters, including knee extension strength, maximum walking speed, and other physical function measures, was greater in the monthly CR group than in the non-monthly CR group.

**Table 2:** Changes in laboratory findings and physical function.

Variables	All patients n = 66	Monthly CR n = 27	Non-monthly CR n = 39	*p* value
***Clinical characteristics***				
ΔBody mass index, kg/m^2^	0.83 ± 3.7	1.4 ± 5.7	0.77 ± 1.2	0.244
ΔMean blood pressure, mmHg	−2.3 ± 10.2	−4.9 ± 12.0	−0.6 ± 8.8	0.095
ΔBlood glucose, mg/dl	0.1 ± 19.8	1.7 ± 11.6	−1.1 ± 23.8	0.558
ΔHemoglobin A1c, %	−0.11 ± 0.27	−0.21 ± 0.31	−0.04 ± 0.22	0.014*
ΔLDL-cholesterol, mg/dL	1.0 ± 7.3	−0.7 ± 6.8	2.3 ± 7.5	0.103
ΔHDL-cholesterol, mg/dL	0.24 ± 6.6	−0.3 ± 8.7	0.6 ± 4.8	0.563
ΔTriglyceride, mg/dL	−6.0 [−26.2−7.0]	−12.0 [−3.0−2.0]	−3.0 [−18.0−8.0]	0.027 *
ΔBrain natriuretic peptide, pg/mL	−5.7 [−38.1−22.9]	−30.6 [−61.4−6.0]	15.6 [−13.8−33.9]	0.015 *
ΔLeft ventricular ejection fraction, %	0.1 ± 1.7	0.3 ± 1.8	0.0 ± 1.7	0.462
ΔE/e′	−0.5 ± 3.4	−1.4 ± 0.7	1.0 ± 0.6	0.007 *
ΔIntima media thickness, mm	−0.1 ± 0.2	−0.1 ± 0.2	0.0 ± 0.2	0.054
ΔPulse wave velocity, cm/s	7.5 ± 134	−22.2 ± 145	28.1 ± 123	0.133
ΔRH-PAT index	0.01 ± 0.25	0.14 ± 0.27	−0.08 ± 0.18	<0.001 *
***Physical functions***				
ΔGrip, kg	−0.2 ± 3.6	−0.2 ± 3.7	−0.2 ± 3.6	0.985
ΔQuadriceps isometric strength, kg	0.0 ± 3.4	2.3 ± 2.8	−1.6 ± 2.7	<0.001 *
ΔWalking speed, m/s	0.0 ± 0.1	20.5 ± 4.1	−8.4 ± 3.3	<0.001 *
ΔOne-leg standing, s	−0.3 ± 13.3	6.1 ± 13.3	−4.7 ± 11.5	<0.001 *
ΔFunctional reach, cm	−0.1 ± 2.8	2.2 ± 0.4	−1.1 ± 0.3	<0.001 *
ΔSix-minute walking distance, m	2.8 ± 17.2	40.0 ± 7.1	−14.1 ± 6.5	<0.001 *

Values are presented as mean ± standard deviation.

* *p* <0.05. CR, cardiac rehabilitation; LDL, low-density lipoprotein; HDL, high-density lipoprotein; E/e′, peak early diastolic velocity/basal septal diastolic velocity ratio; RH-PAT, reactive hyperemia peripheral arterial tonometry.

### Comparison of cognitive function change between the monthly CR and non-monthly CR groups

**Fig 1:**
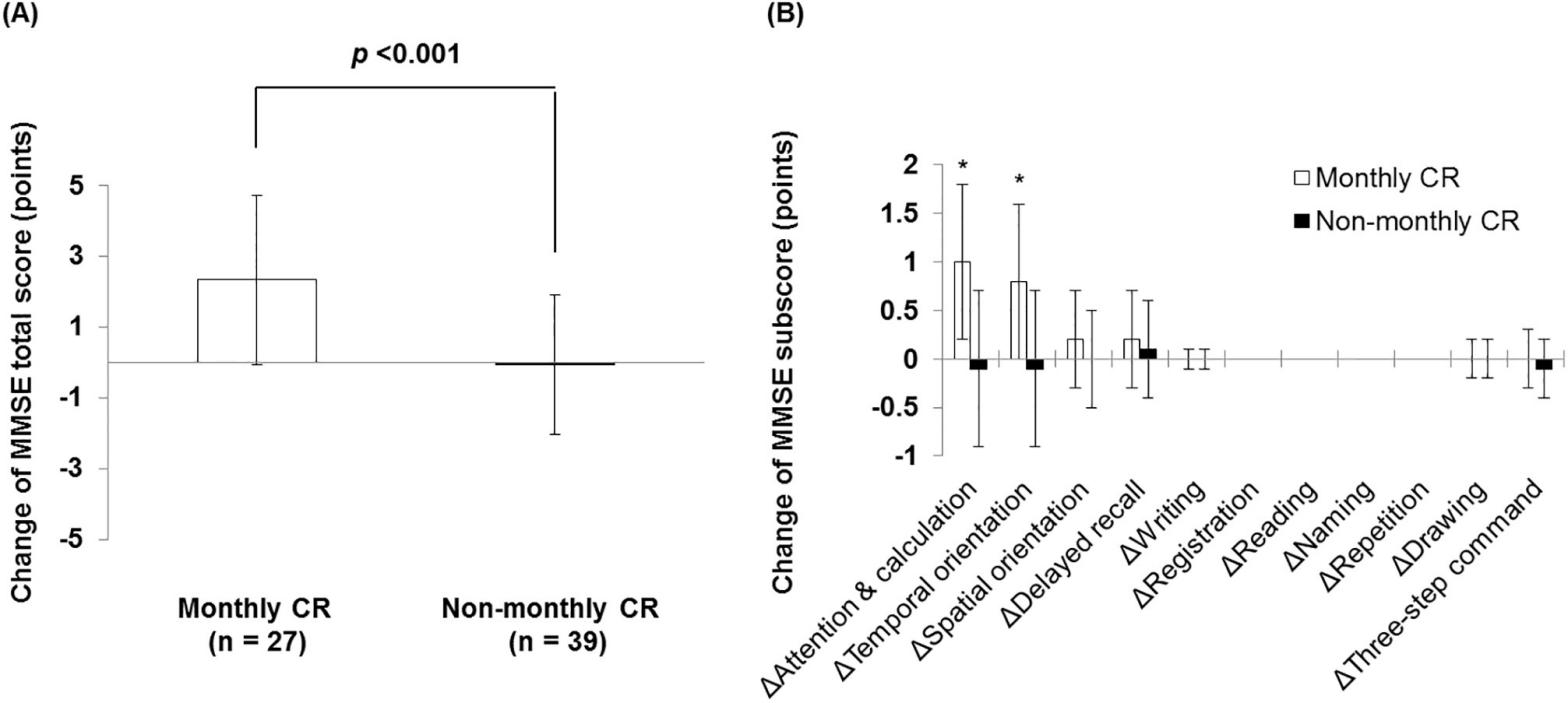
Change in the MMSE score during 6 months. (A) Change in the MMSE total score for 6 months between the monthly CR group and the non-monthly CR group. (B) Changes in the MMSE items for 6 months between the monthly CR group and the non-monthly CR group. CR, cardiac rehabilitation; MMSE, Mini-mental State Examination.

**Fig 2:**
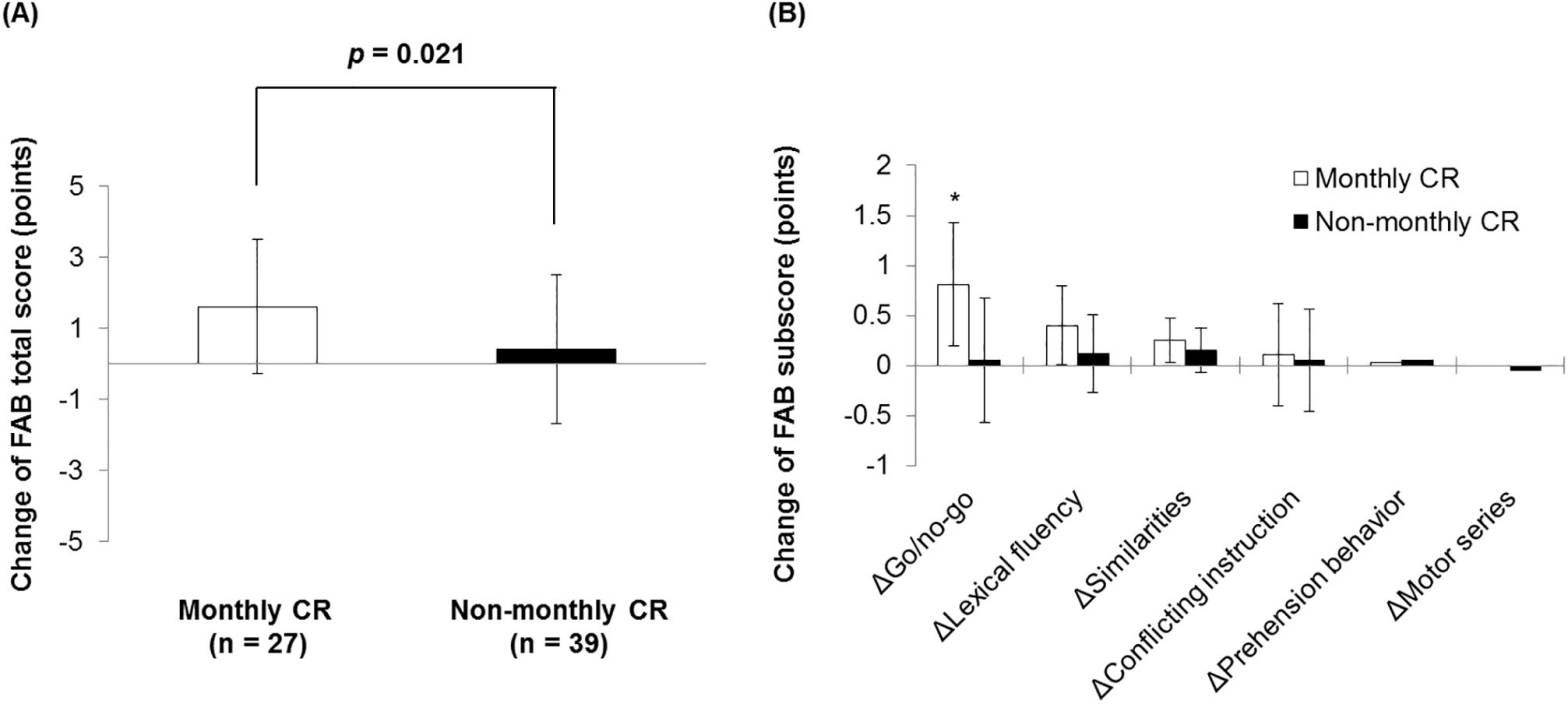
Change in the FAB score during 6 months. (A) Change in the FAB total score during 6 months between the monthly CR group and the non-monthly CR group. (B) Changes in FAB items during 6 months between the monthly CR group and the non-monthly CR group. CR, cardiac rehabilitation; FAB, Frontal Assessment Battery.

The change in the MMSE score for 6 months was greater in the monthly CR group than in the non-monthly CR group (−0.1 ± 0.3 vs. 2.3 ± 0.4 points; *p* <0.001; Fig 1A). The change in orientation for time and computing power was also greater in the monthly CR group than in the non-monthly CR group (Fig 1B). The change in the FAB score was also greater in the monthly CR group than in the non-monthly CR group (0.4 ± 2.1 vs. 1.6 ± 1.9 points; *p* = 0.021; Fig 2A). The change in the Go/No-Go task of the FAB was also greater in the monthly CR group than in the non-monthly CR group (Fig 2B). The comparisons of change in MMSE and FAB between the monthly CR group and the non-monthly CR group according to the baseline MMSE categories are shown in S2 Fig (S2 Fig, S1 Table in S1 File).

### Independent predictors for changes in cognitive function

**Fig 3:**
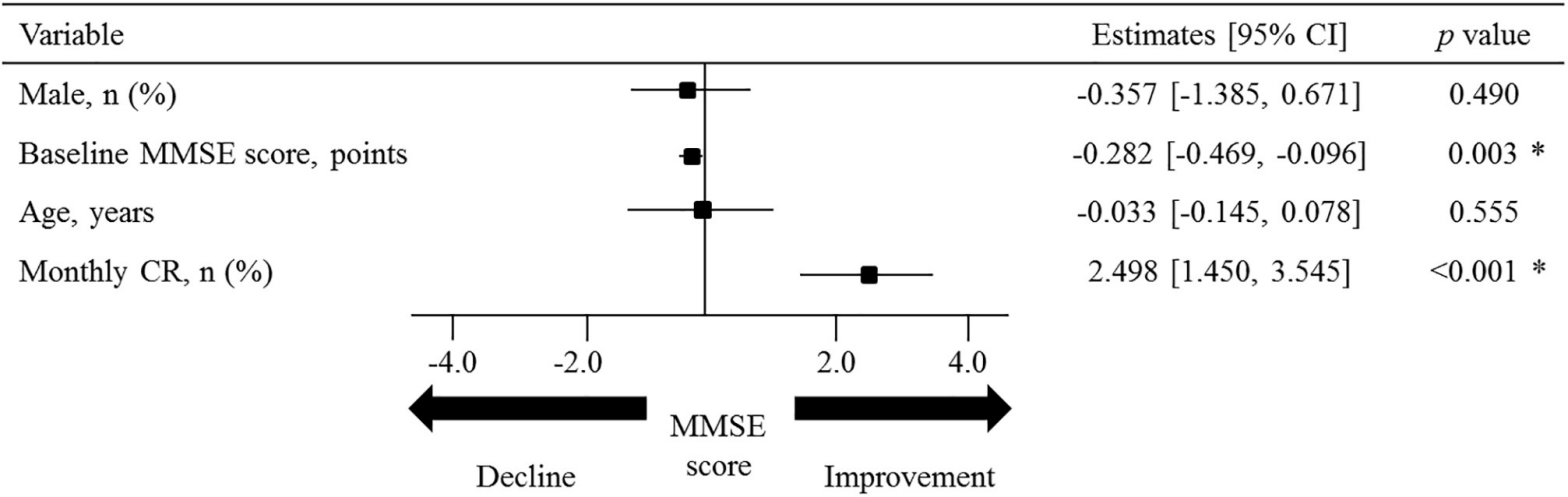
Clinical factors for the response of the MMSE score. General linear modeling analysis for the absolute change in the MMSE score shows that the baseline MMSE score and monthly CR are predictors of unfavorable and favorable response, respectively. CI, confidence interval; CR, cardiac rehabilitation; MMSE, Mini-mental State Examination.

The general linear model revealed that monthly CR (effect estimate, 1.455; 95% confidence interval [CI], 0.747–2.163; *p* <0.001) was an independent predictor for the absolute change in the MMSE score (Fig 3). Similar results were obtained in the analysis for the FAB score (S3 Fig).

### Correlation between the number of CR and changes in cognitive function

**Fig 4:**
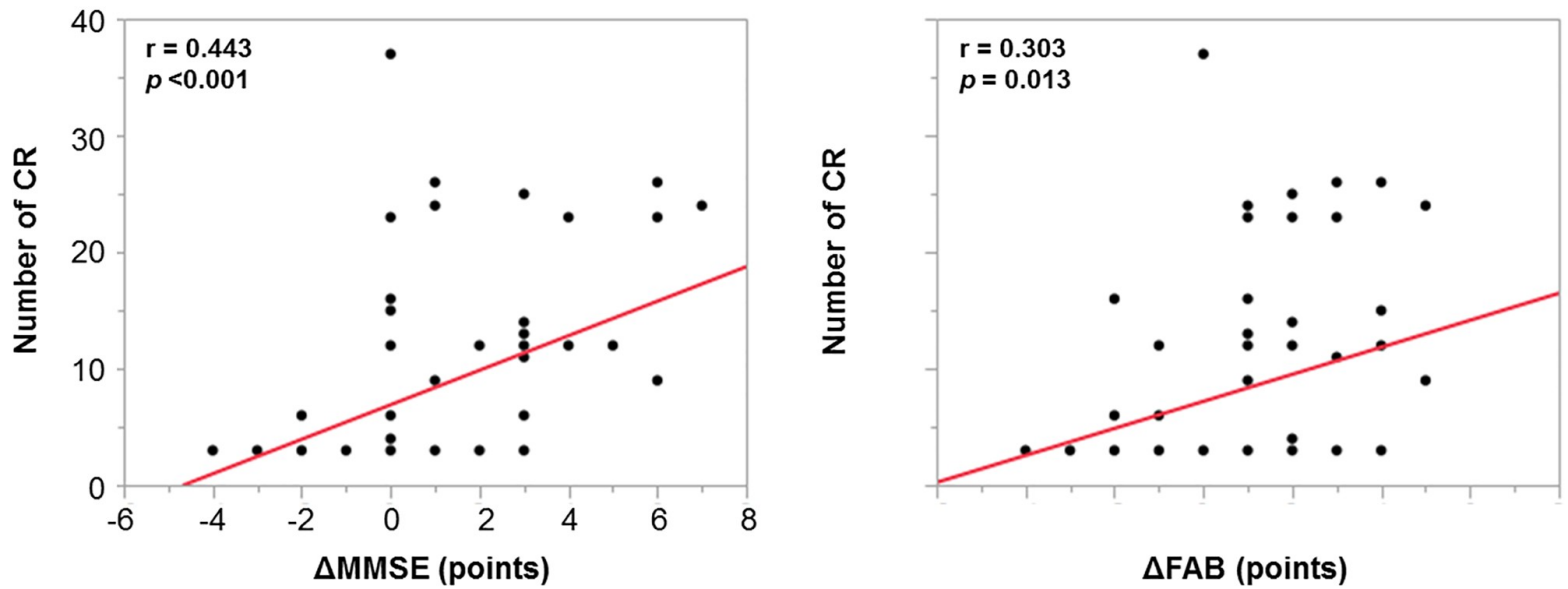
Correlation between the number of CR and change in cognitive function. A significant linear correlation was observed between the number of CR and the change in cognitive function assessed using MMSE and FAB. CR, cardiac rehabilitation; FAB, Frontal Assessment Battery; MMSE, Mini-mental State Examination.

Significant correlations were observed between the number of CR and the change in the MMSE score (Pearson r = 0.443, *p* <0.001) and the FAB score (Pearson r = 0.303, *p* = 0.013) (Fig 4).

### ROC analysis for the number of CR needed to detect cognitive improvement

**Fig 5:**
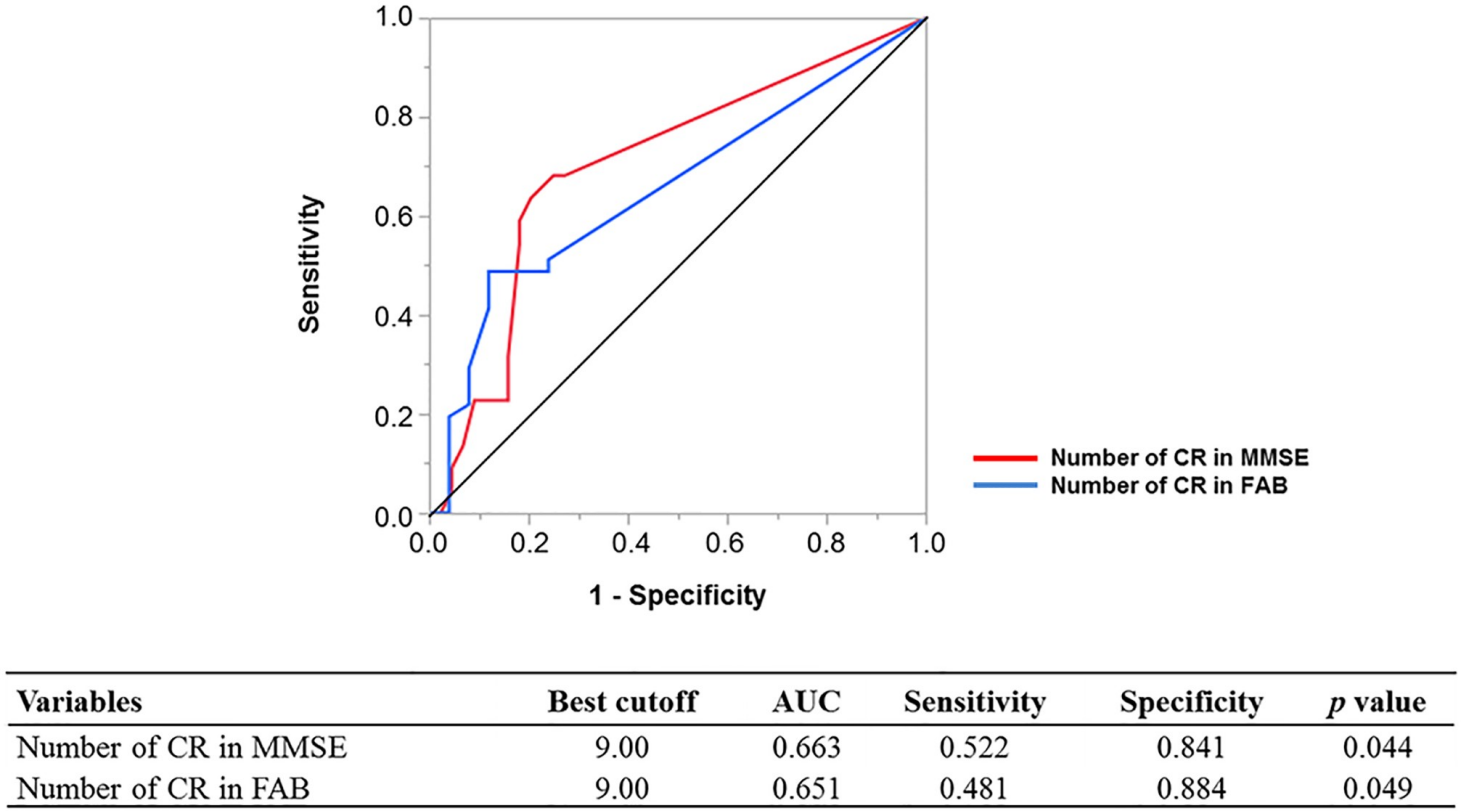
Receiver-operating characteristic curve to detect cognitive improvement. AUC, area under curve; CR, cardiac rehabilitation; FAB, Frontal Assessment Battery; MMSE, Mini-mental State Examination.

The area under the ROC curve for the detection of cognitive improvement assessed using MMSE and FAB was 0.66 (95% CI, 0.42–0.89; *p* = 0.044) and 0.65 (95% CI, 0.53–0.95; *p* = 0.049), respectively (Fig 5). The best cutoff value of the number of CR for the detection of cognitive improvement assessed using the MMSE and FAB was 9.0 and 9.0, respectively.

## Discussion

The main findings of the present study were as follows: 1) the improvement of cognitive function was significantly greater in the monthly CR group than in the non-monthly CR group; 2) among cognitive functions, the improvement of frontal cortical function was significantly greater in the monthly CR group than in the non-monthly CR group; and 3) the improvement of laboratory parameters related to arteriosclerotic risks, cardiac function, and physical abilities were also greater in the monthly CR group than in the non-monthly CR group. The results of the present study suggest that regular CR may help improve cognitive function in addition to physical function in elderly patients with CVD.

### Efficacy of CR in improving cognitive function

Previous studies have demonstrated the efficacy of comprehensive rehabilitation including cognitive function training on the maintenance and improvement of cognitive function. [16, 17] In a randomized controlled trial, Kwak et al. demonstrated that program-based regular exercise (30–60 min/day, 2–3 times per week for 12 months) improved the MMSE score from 14.5 ± 5.3 to 17.5 ± 6.9 points in patients with severe cognitive impairment. [18] Ngandu et al. demonstrated the efficacy of 2-year multidomain intervention including regular exercise and cognitive training on the improvement of cognitive function evaluated using a neuropsychological test battery in elderly participants with dementia in a randomized trial. [4] On the other hand, the present study demonstrated the efficacy of a CR program without cognitive training on the improvement of MMSE and FAB scores. Although the exact causal mechanisms of the efficacy of CR in improving cognitive function are still undetermined, we believe that 3 factors potentially contributed to the improvement of cognitive function by CR.

First, the improvement of endothelial function might contribute to the improvement of cognitive function. Our group recently reported a significant association between the presence of endothelial dysfunction evaluated using RH-PAT and impaired cognitive function. [2] In the present study, we further reported that the change in the RH-PAT index was greater in the monthly CR group than in the non-monthly CR group. The improvement of endothelial function may contribute to the recovery or maintenance of cognitive function through improvements in the cerebral microcirculation via enhanced nitric oxide synthesis, prostacyclin, tissue plasminogen activator, and shear stress. [19]

Second, the improvement of cardiac function might also contribute to the improvement of cognitive function. [20, 21] Although the direct influence of cardiac function on the improvement of cognitive function is still unclear, the significant correlations between the 2 factors were demonstrated in several previous studies. Jefferson et al. investigated a cohort in the Framingham Heart Study and reported that low left ventricular ejection fraction (<62.0%) was associated with low cognitive performance in patients with CVD. [22] Sauvé et al. also reported that the odds of cognitive impairment were 4.47 greater in elderly patients with heart failure than in healthy controls. [23] In the present study, the monthly CR group showed greater reduction in brain natriuretic peptide levels and greater recovery of left ventricular ejection fraction, in addition to the significant improvement of cognitive function, than the non-monthly CR group.

Third, the improvement of physical function may also contribute to the improvement of cognitive function. Verghese et al. reported that the gait velocity was significantly lower in elderly persons with cognitive impairment than in those with healthy cognitive function. [24] Another study reported that gait abnormalities including slow gait confer a greater risk for the development of dementia (hazard ratio, 1.96) in the elderly. [25] In the present study, improvements in maximum walking speed, knee extension strength, 1-leg standing, and functional reach, which are related to skeletal muscle mass and balance function, were observed. This resulted in an improvement in the 6-min walking distance, which is related to exercise tolerance. [26] Presumably, these comprehensive effects of CR may improve cognitive function without cognitive training.

### Efficacy of CR in improving frontal cortical function

A previous study reported that a comprehensive rehabilitation program including cognitive training improved the frontal cortical function including executive function and processing speed. [4, 27] The present study also demonstrated that monthly CR significantly improved the frontal cortical function. However, the reason for the improvement in specific functions after rehabilitation programs remains unclear. Although increase in whole cerebral blood flow after exercise therapy was reported, [28] the increase of cerebral flow in a specific cortex after rehabilitation programs has not been demonstrated thus far. In addition to the exercise itself, scheduled visits to the rehabilitation site and conversations with rehabilitation staff might have stimulatory effects on the functions of the frontal cortex. [29]

### Limitations

Several limitations of this study need to be acknowledged. First, this was an observational study with a small-sized specific cohort from a single center. Thus, it might be difficult to generalize the results. Further studies with a randomized design are required to further clarify the impact of CR on cognitive functions. Second, the type of CVD in the present cohort varied. The impact of CR on each disease remains undetermined. Third, the study period was only 6 months. A longer follow-up may further clarify the relevance of CR in changing the cognitive functions. Fourth, the causal relationship between CR and cognitive improvement was not clarified by the findings of this observational study.

## Conclusions

In elderly patients with CVDs, regular cardiac rehabilitation had a potential to improve cognitive function. The present results may partly explain the efficacy of cardiac rehabilitation for secondary prevention. Further studies with a randomized design are required to further clarify the impact of CR on the improvement of cognitive functions.

## Supporting information

S1 Fig Study flow chart. CR, cardiac rehabilitation.
(TIF)

S2 Fig Comparisons of change in MMSE and FAB between the monthly CR group and the non-monthly CR group according to the baseline MMSE categories. MMSE, Mini-mental State Examination; FAB, Frontal assessment battery; CR, cardiac rehabilitation.
(TIF)

S3 Fig Clinical predictors for the response of FAB score. General linear modeling analysis for the absolute change in the FAB score shows that the baseline FAB score and monthly CR are predictors of unfavorable and favorable response, respectively. CI, confidence interval; CR, cardiac rehabilitation; FAB, Frontal assessment battery.
(TIF)

S1 File Supplementary methods. Assessment of endothelial function, Measurement of physical functions, Definition; **Supplementary table.** S1 Table. Comparisons of baseline and change in cognitive functions according to the baseline MMSE categories; **Supplementary references.**
(DOCX)

S1 Database (XLSX)
