# Supplementary figures and images for: Effect of cardiac rehabilitation on cognitive function in elderly patients with cardiovascular diseases
Source: PLoS One. 2020 May 29;15(5):e0233688. doi: 10.1371/journal.pone.0233688 (PMC7259633; doi:10.1371/journal.pone.0233688)

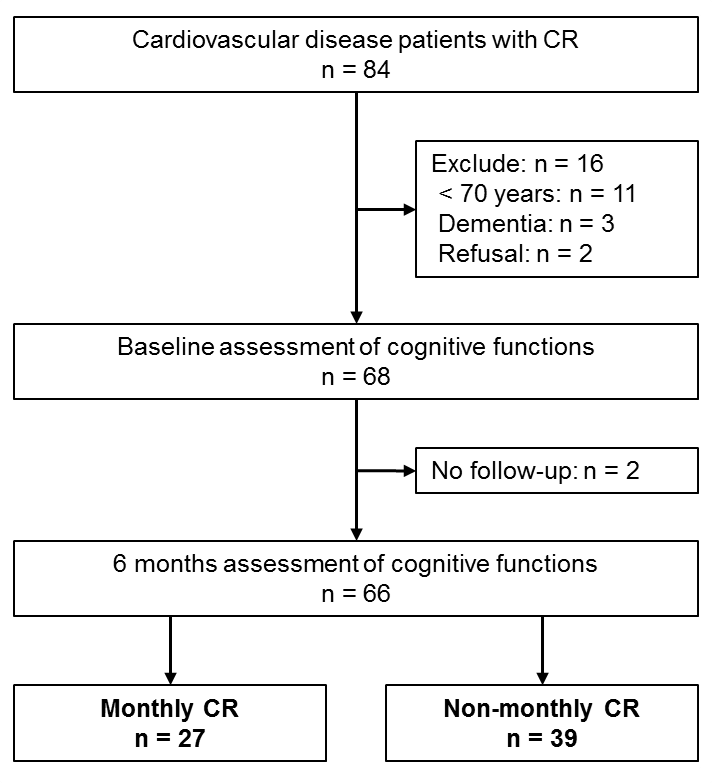

Supplement: S1 Fig — CR, cardiac rehabilitation. (TIF) [file pone.0233688.s001.tif]

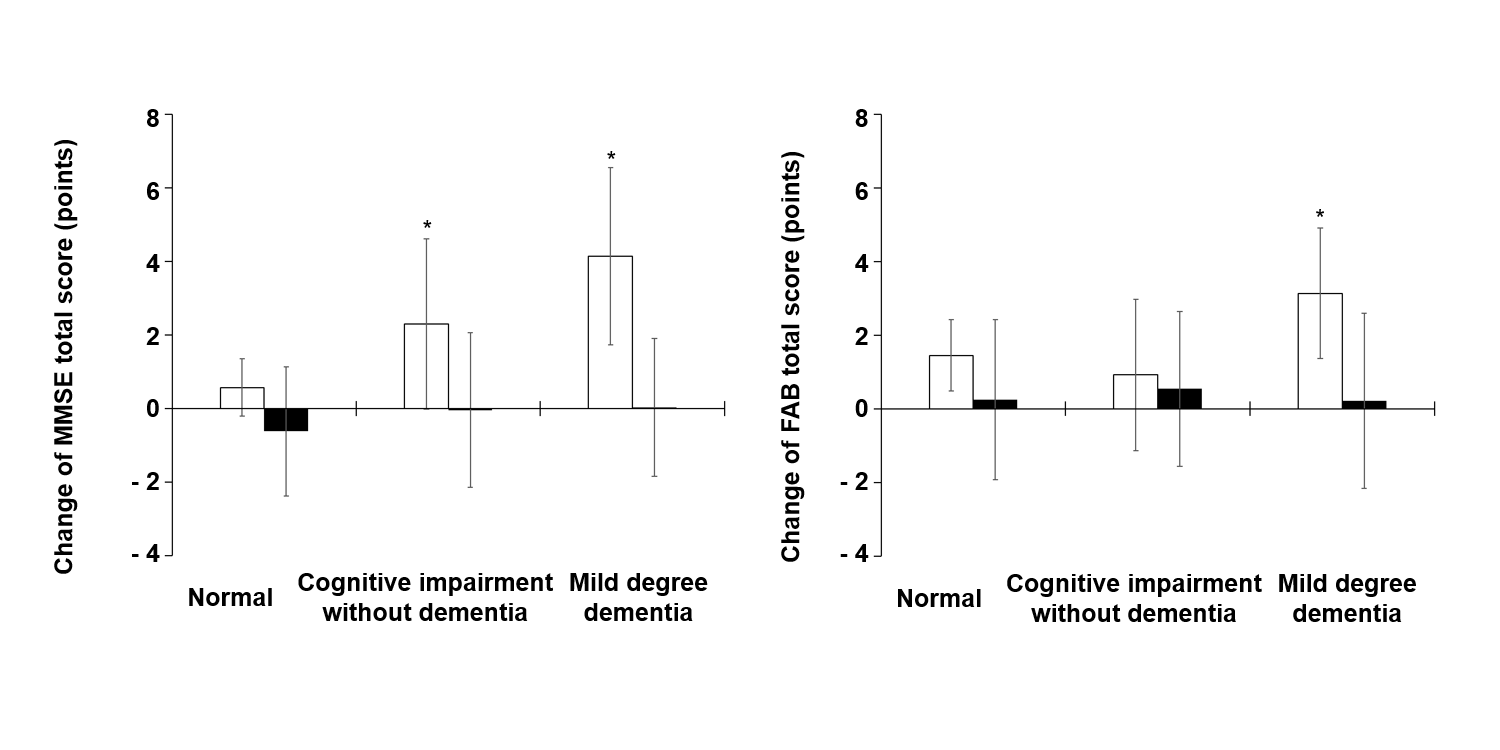

Supplement: S2 Fig — MMSE, Mini-mental State Examination; FAB, Frontal assessment battery; CR, cardiac rehabilitation. (TIF) [file pone.0233688.s002.tif]

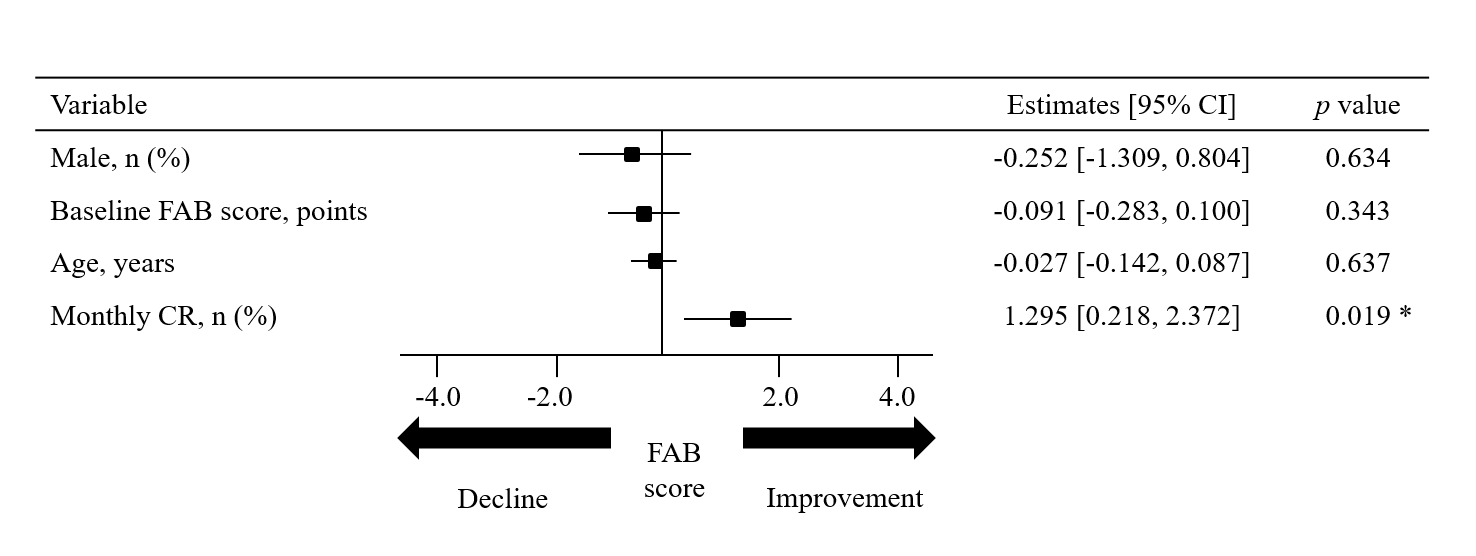

Supplement: S3 Fig — General linear modeling analysis for the absolute change in the FAB score shows that the baseline FAB score and monthly CR are predictors of unfavorable and favorable response, respectively. CI, confidence interval; CR, cardiac rehabilitation; FAB, Frontal assessment battery. (TIF) [file pone.0233688.s003.tif]
