# Supplementary material for: Effect of cardiac rehabilitation on cognitive function in elderly patients with cardiovascular diseases
Source: PLoS One. 2020 May 29;15(5):e0233688. doi: 10.1371/journal.pone.0233688 (PMC7259633; doi:10.1371/journal.pone.0233688)
Supplement: S1 File — Assessment of endothelial function, Measurement of physical functions, Definition; Supplementary table. S1 Table. Comparisons of baseline and change in cognitive functions according to the baseline MMSE categories; Supplementary references. (DOCX) [file pone.0233688.s004.docx]

**SUPPLEMENTARY MATERIALS**

**Effect of cardiac rehabilitation on cognitive function in elderly patients with cardiovascular diseases**

Kazuhiro Fujiyoshi, MD, PhD;^1^ Yoshiyasu Minami, MD, PhD;^1^ Minako Yamaoka-Tojo, MD, PhD;^2^* Toshiki Kutsuna, PhD;^3^ Shinichi Obara, MSc;^3^ Akihiro Aoyama, MSc;^2^ and Junya Ako, MD, PhD^1^

1. Department of Cardiovascular Medicine, Kitasato University School of Medicine, Sagamihara, Japan

2. Department of Rehabilitation, Kitasato University School of Allied Health Sciences, Sagamihara, Japan

3. Department of Cardiac Rehabilitation, Kitasato University East Hospital, Sagamihara, Japan

**Supplementary methods**

**Supplementary table**

**Supplementary references**

**Supplementary** **methods**

**Assessment of endothelial function**

The endothelial function was evaluated by RH-PAT (EndoPAT2000; Itamar Medical, Caesarea, Israel).^1, 2^ On the day of visit, the patients were not allowed any caffeine-containing drinks or tobacco consumption. Before measurement, the patients were asked to rest for 10 minutes. In line with previous studies, we applied 10 minutes’ rest before the measure- ment.^3, 4^ Using a fingertip peripheral arterial tonometry device, we measured the digital pulse amplitude in the supine position of the patients for 5 minutes at baseline and after a reactive hyperemia induced by a 5-minute forearm cuff occlusion. RH-PAT was measured in a fasting condition. The data were digitized and were computed automatically with EndoPAT2000 software. The digital RH-PAT index, representing the endothelial function, was defined as the ratio of the mean post-deflation signal (in the 90 to 120-s post-deflation interval) to the baseline signal in the hyperemic finger and was normalized by the same ratio in the contra-lateral finger and was multiplied by a baseline correction factor, as calculated by the EndoPAT 2000 software. An independent clinical physician, who was blinded to the outcomes of the cognitive function performed the RH-PAT evaluation

**Measurement of physical functions**

Physical functions were measured in below. To measure walking speed, the patients were asked to walk at their usual speed, and were timed over the middle 10 m of a 16-m walkway.^5^ To measure handgrip strength, a digital dynamometer (TKK 5101 Grip-D; Takei, Tokyo, Japan) was used with the patient in the sitting position. Two maximal isometric voluntary contractions of the hands for 3 s each were collected for both hands. The average of the maximum value of the both sides was used for the analysis.^5^ To measure one-leg standing time, the patients were required to stand on one leg for up to 60 s with the hands placed on the hips and the eyes open. The patients performed the test twice, and the best time obtained was recorded.^6^ To measure functional reach, patients stood with feet apart at shoulder width, and hands beside the body. Location of the third metacarpal bone was recorded as a starting point. They reached forward as far as possible without taking a step. The ending point was recorded. Distance between starting and ending points was recorded twice. Reaching distance was the averaged value presented in cm.^7^

**Definition**

Hyperlipidemia was defined as high-density lipoprotein cholesterol < 40 mg/dL, low-density lipoprotein cholesterol > 140 mg/dL, or triglycerides > 150 mg/dL or taking medication for dyslipidemia. Diabetes mellitus was defined as symptoms of diabetes plus casual plasma glucose concentration > 200 mg/dL, fasting plasma glucose concentration > 126 mg/dL, 2hours plasma glucose concentration > 200 mg/dL during 75 g oral glucose tolerance test, or taking medication for diabetes mellitus.

**Supplementary table**

**Supplementary Table 1. Comparisons of baseline and change in cognitive functions according to the baseline MMSE categories**

| **Variables** | **Normal**  **n = 15** | **Cognitive impairment without dementia**  **n = 35** | **Mild degree dementia**  **n = 16** | ***p* value** |
| --- | --- | --- | --- | --- |
| Baseline cognitive functions |  |  |  |  |
| MMSE, points | 29.0 ± 2.8 | 25.3 ± 1.1 | 21.7 ± 1.6 | < 0.001 * |
| FAB, points | 15.2 ± 1.4 | 13.7 ± 2.0 | 11.5 ± 1.5 | < 0.001 * |
| Change of cognitive functions |  |  |  |  |
| ΔMMSE, points | −0.06 ± 1.48 | 0.82 ± 2.4 | 2.0 ± 2.8 | 0.057 |
| ΔFAB, points | 0.08 ± 1.78 | 0.68 ± 2.0 | 1.5 ± 2.5 | 0.444 |

Values were represented by mean ± SD, or n (%). * *p* value < 0.05. MMSE, mini-mental statement examination. FAB, frontal assessment battery.

**Supplementary references**

1. Kuvin JT, Patel AR, Sliney KA, et al. Assessment of peripheral vascular endothelial function with finger arterial pulse wave amplitude. *Am Heart J* 2003; 146: 168–174.

2. Hamburg NM, Keyes MJ, Larson MG, et al. Cross-sectional relations of digital vascular function to cardiovascular risk factors in The Framingham Heart Study Naomi. *Circulation* 2009; 117: 2467–2474.

3. Matsubara J, Sugiyama S, Akiyama E, et al. Dipeptidyl peptidase-4 inhibitor, sitagliptin, improves endothelial dysfunction in association with its anti-inflammatory effects in patients with coronary artery disease and uncontrolled diabetes. *Circ J* 2013; 77: 1337–44.

4. Liu J, Wang J, Jin Y, et al. Variability of peripheral arterial tonometry in the measurement of endothelial function in healthy men. *Clin Cardiol* 2009; 32: 700–704.

5. Tanaka S, Kamiya K, Hamazaki N, et al. Incremental Value of Objective Frailty Assessment to Predict Mortality in Elderly Patients Hospitalized for Heart Failure. *J Card Fail* 2018; 24(11): 723-32.

6. Matsuzawa R, Kamiya K, Hamazaki N, et al. Office-Based Physical Assessment in Patients Aged 75 Years and Older with Cardiovascular Disease. *Gerontology.* 2019; 65(2): 128-35.

7. Nnodim JO, Yung RL, et al. Balance and its Clinical Assessment in Older Adults - A Review. *J Geriatr Med Gerontol* 2015; 1(1): 003.
